# Integrating Salutogenesis into Wellness in Every Stage of Life

**Published:** 2007-06-15

**Authors:** Jane Ellery

**Affiliations:** Fisher Institute for Wellness and Gerontology, Ball State University, Muncie, Indiana

## To the Editor:

I enjoyed reading Dr. Steinberg's editorial "Wellness in Every Stage of Life: A New Paradigm for Public Health Programs" (1) and the related articles in the January 2007 issue. We face many challenges in conceptualizing wellness because the term has different meanings for different people. To reach a common understanding of *wellness*, we could begin by using Antonovsky's salutogenesis work (2-4) as a basis for discussion. Using salutogenesis (origins of health) as a model for public health practice rather than pathogenesis (origins of disease) allows us to focus on factors that support and increase well-being rather than on factors that merely prevent disease. Antonovsky suggests that people feel a *sense of coherence* if their lives are meaningful, manageable, and comprehensible, which suggests that an orderly and consistent life is more desirable than a chaotic, random, and unpredictable life. Therefore, when deciding how to bring about beneficial behavior change, we must consider genetic, constitutional, and psychosocial factors such as people's level of knowledge and intelligence as well as their ego identity, coping strategy, social support system, religion, philosophy, and attitude toward health maintenance.

When we look at people from a multilevel perspective, as encouraged by the Institute of Medicine's report *Who Will Keep the Public Healthy?* (5), we find that interpersonal factors, sociocultural factors, public policies, and physical environment (6) all affect how people perceive their lives and use the resources available to them. We must consider each individual's perception of the world when we design programs to improve well-being. However, in public health practice, we cannot design programs for specific individuals. We work to improve the well-being of populations and must therefore rely on data to give us an understanding of the populations for whom we intend to create programs. When we learn about the population's most common perceptions, passions, knowledge, attitudes, and behaviors, we can create products and offer services that will improve people's sense of coherence.

Public health can work well from a salutogenesis perspective, although the underlying philosophy of salutogenesis is different from the usual disease-prevention and risk-avoidance practices in use. Research methods, planning, and evaluation are similar for many behavior-change strategies, but the thought processes involved in strategizing from a salutogenesis perspective are different from those involved in strategizing from a pathogenesis perspective. Not better or worse, just different. A salutogenic focus changes how we see issues related to health and well-being. Instead of developing solutions based on decreasing health-related risks, we find ways to promote healthful behaviors that increase people's sense of well-being, their sense of coherence.

So, what does all of this mean? It means we must listen to people in the communities we want to serve to learn about their areas of concern, and we must recognize that people's life experience is an important factor in whether they are willing to change and what will inspire them to do so.  More than likely, interventions taking a salutogenic approach will

- Limit emphasis on clinical health risks (e.g., hypertension, elevated cholesterol).
- Focus initially on improving morale within communities and developing meaningful relationships between community members and public health practitioners.
- Emphasize personal changes (e.g., increasing activity levels, controlling body weight, decreasing stress and anxiety) and community changes (e.g., creating parks and bike paths, adopting no-smoking policies) to increase people's opportunities to lead healthful lifestyles.
- Include mental health as a key factor in improving people's well-being, with an emphasis on changing attitudes, increasing self-knowledge, becoming responsible, and being empowered.
- Emphasize programs that are fun rather than competitive or difficult (e.g., non-competitive games, fun runs, walking programs, nutritional potlucks).

We must expand current practices associated with health care delivery, disease control and prevention, risk reduction, and health promotion to include wellness management. Doing so will expand the opportunities available to us as we work together to improve the health of our nation. I am excited that CDC is embracing wellness as part of public health practice.
